# Investigating the utilization of radiological services by physician patients: a population-based cohort study in Taiwan

**DOI:** 10.1186/1472-6963-13-284

**Published:** 2013-07-23

**Authors:** Chen-Yi Wu, Hsiao-Yun Hu, Likwang Chen, Nicole Huang, Yiing-Jeng Chou, Chung-Pin Li

**Affiliations:** 1 Institute of Public Health, National Yang-Ming University, No. 155, Sec. 2, Li-Nong Street, Taipei, Taiwan; 2 Division of Dermatology, Heping Fuyou Branch, Taipei City Hospital, No. 33, Sec. 2, Chung-Hwa Road, Taipei, Taiwan; 3 Department of Education and Research, Taipei City Hospital, No. 145, Zhengzou Road, Taipei, Taiwan; 4 Division of Health Policy Research and Development, The Institute of Population Health Sciences, National Health Research Institutes, Miaoli County, Taiwan; 5 Institute of Hospital and Health Care Administration, National Yang-Ming University, No. 155, Sec. 2, Li-Nong Street, Taipei, Taiwan; 6 Division of Gastroenterology, Department of Medicine, Taipei Veterans General Hospital, No. 201, Sec. 2, Shih-Pai Road, Taipei 11217, Taiwan; 7 National Yang-Ming University School of Medicine, No. 155, Sec. 2, Li-Nong Street, Taipei, Taiwan

**Keywords:** Utilization, Radiology service, Profession, CT, MRI

## Abstract

**Background:**

Advances in radiology technology have contributed to a substantial increase in utilization of radiology services. Physicians, who are well educated in medical matters, would be expected to be knowledgeable about prudent or injudicious use of radiological services. The aim of this study was to evaluate differences in the utilization of radiology modalities among physician and non-physician patients.

**Methods:**

This nationwide population-based cohort study was carried out using data obtained from the Taiwan National Insurance Database from 1997 to 2008. Physicians and comparison controls selected by propensity score matching were enrolled in the current study. The claims data of ambulatory care and inpatient discharge records were used to measure the utilization of various radiology modalities. Utilization rates of each modality were compared between physicians and non-physicians, and odds ratios of the utilization of each radiology modality were measured. Multiple logistic regression analysis was used to examine the predictors of X-ray, MRI, and interventional procedures utilization during the study period.

**Results:**

The utilization of most radiologic services increased among physicians and the comparison group during the observation period. Compared to non-physicians, physicians had significantly higher utilization rates of computed tomography and magnetic resonance imaging (MRI) but lower utilization rates of X-rays, sonography, and interventional procedures. After adjusting for age, gender, major diseases, urbanicity, and residential regions, logistic regression analysis showed that, compared to non-physicians, the physicians used significantly more MRI (odds ratio [OR]: 2.19, 95% confidence interval [CI]: 1.68–2.84, *P* < 0.001) and significantly less X-rays and interventional procedures (OR: 0.85, 95% CI: 0.72–0.99, *P* = 0.04 for X-rays and OR: 0.67, 95% CI: 0.54–0.83, *P* < 0.001 for interventional procedures). Being a physician was a significant predictor of greater usage of MRI and of less usage of X-ray and interventional procedures.

**Conclusions:**

This study revealed different utilization patterns of X-rays, MRI, and interventional procedures between physician and non-physician patients, even after controlling for such factors as socioeconomic status and major diseases.

## Background

Rapid advances in radiology procedures have greatly enhanced the ability of physicians to diagnose and treat a variety of diseases. However, these technological improvements have led to increases in use of and expenditures associated with radiology services [1-3]. Much of the rapid growth in radiology procedure volume is attributable to the more advanced examinations, such as computed tomography (CT), magnetic resonance imaging (MRI), and positron-emission tomography (PET) [4].

The large increase in use of radiology services in recent decades has had a significant influence on health care costs, quality of health care, and individual health status. However, whether radiology procedures are overused, underused, or misused has been debated for decades, and this discussion has become more imperative as expenditures associated with radiology procedures continue to rise despite medical care resource constraints. The appropriate use of radiology services is difficult to define, since an objective measure to assess the value of radiology procedures in terms of improved health care outcomes is currently lacking [4-6].

The National Health Insurance (NHI) program in Taiwan provides mandatory universal health insurance and offers comprehensive medical care coverage to all civilian Taiwanese residents. It has removed barriers to medical care and enabled more equal access to health care. Conventional X-rays, sonography, CT, and interventional procedures have been available under the universal health insurance coverage since 1997, and MRI, radioisotope scanning, and PET have been available since 2004. For radiology modalities covered by insurance in ambulatory or inpatient care, patients pay only a minimal user fee and copayment. NHI rules exist for PET utilization, with use approved only for some specific cancer patients [7]. Under NHI, patients have unlimited free choice of physicians and health care facilities. There is no mandated physician referral system or coordinated system of health care delivery in Taiwan [8]. Every physician in charge has the right to prescribe radiological examinations.

Many previous studies have explored the great increase in utilization of radiology procedures, and the role of physicians as providers has been investigated [9-11]. Physicians, as experts in medical matters, are expected to understand the proper use of radiological services and to understand the problems associated with injudicious use. Thus, the utilization pattern of radiologic services of physicians who are also patients would be an interesting issue to explore. The objective of this study was to investigate whether and to what extent utilization of radiological services differs for physician patients in comparison to general adults after the removal of financial barriers to treatment under a national health insurance program.

## Methods

### Data source

The NHI is a mandatory universal health insurance program offering comprehensive medical care coverage to all Taiwanese residents. Ninety-six percent of residents in Taiwan have joined the NHI program since 1996. The NHI sample files, constructed and managed by the National Health Research Institutes, consist of comprehensive utilization and enrollment information for a randomly selected sample of 1,000,000 NHI beneficiaries, representing approximately 5% of all enrollees in Taiwan in 2005 [12]. A multistage stratified systematic sampling design was used. There were no statistically significant differences in age or gender between the sample groups and all enrollees. The comprehensive health care data include enrollment files, claims data, major disease files, medical personnel registry, and a registry for drug prescriptions. All information that would allow a specific patient to be identified is encrypted. The confidentiality of the data is enforced through the data regulations of the Bureau of National Health Insurance, Taiwan. The current study was approved by the National Health Research Institutes and the Institutional Review Board of Taipei Veterans General Hospital and was conducted in accordance with the Helsinki Declaration.

### Study population

We conducted a retrospective cohort study covering the period from January 1, 1997, to December 31, 2008, using the 1,000,000 NHI sample files described above. In order to classify patients as physicians or general adults, we used the NHI medical personnel and household registries to identify persons with “physician” as their recorded occupation. Since people rarely qualify as physicians before 25 years of age, and because there were limited numbers of comparison cases above age 65, we excluded subjects below 25 and above 65 years of age.

### Comparison group

Since the socioeconomic status (SES) of physicians may be higher than that of general adults, to assure better comparability we included as a pre-matched sample group only those adults aged 25–65 years who had an SES similar to the physicians. The NHI enrollment files provide information on the insurable wages and occupations of the insured. Therefore, SES was inferred by linking patient identifiers and birth dates to the NHI enrollment files. We included in our pre-matched control group only those adults who were regular wage earners, such as civil servants, government employees, private sector employees, teachers, employers, and professionals, with an insurable monthly wage of New Taiwan Dollar 40,000 or greater (equivalent to USD 1,300 in 2008). The pre-matched sample contained 81,627 individuals.

Although comparability of the groups was sought to a satisfactory extent, the question remained of whether any differences between groups might be caused by the huge difference in group sizes. Therefore, nearest-neighbor matching of 1,686 non-physicians to the 1,686 physicians was performed by propensity score technique [13].

### Control variables

Age, gender, major diseases, urbanicity, and residential regions were used as control variables. The diseases were the 30 major diseases or injury types in the NHI major disease list [14], including cancer, end-stage renal disease, autoimmune diseases, chronic psychotic disorders, organ transplantation, and cerebrovascular diseases. In Taiwan, individuals diagnosed with major diseases can apply for a major disease card. Cardholders are exempted from the cost sharing required under the NHI program. Study subjects were also classified into three levels of urbanicity (urban, suburban, and rural) and four geographical regions (north, central, south, and east Taiwan) on the basis of information in the enrollment files.

### Measurements

The claims data of ambulatory care and inpatient discharge records were used to measure utilization of imaging modalities and therapeutic procedures. The claims files include a specific code for each diagnostic and therapeutic radiological procedure performed [7]. The data were grouped according to modalities, and seven main components were measured: conventional X-rays, sonography, CT, MRI, radioisotope scanning, PET, and interventional procedures. Interventional procedures included all image-guided procedures (such as angiography, bronchography, myelography, percutaneous transhepatic cholangiography, endoscopic retrograde cholangiopancreatography, biopsy, drainage, aspiration, etc.). Procedures in which radiation was specifically delivered for therapeutic purposes, such as high-dose radiation therapy for cancers, were excluded. The utilization rates of each modality were measured as the number of procedures per 1,000 person-years throughout the period from 1997 to 2008.

### Statistical analysis

Propensity score technique with nearest-neighbor matching using the demographic variables of age, gender, major diseases, urbanicity, and residential regions were used to select the post-matched controls [13]. Chi-square testing was used to examine the differences in characteristics distributions between physicians and the pre-matched and post-matched comparison groups. Utilization rates of each modality were compared between the two groups. After adjusting for age, gender, major diseases, urbanicity, and residential regions, odds ratios of the utilization of the seven radiology modalities were measured. Multiple logistic regression analysis was used to examine the predictors of X-ray, MRI, and interventional procedures utilization during the study period. We used SAS 9.1 (SAS Institute Inc., Cary, NC, USA) to link the data, and Stata 10 (Stata Corporation, College Station, TX, USA) to perform the statistical analyses.

## Results

### Demographic data

Table 1 shows the baseline characteristics of the 1,686 physicians and the comparison groups of 81,627 pre-matched and 1,686 post-matched samples. Physicians differed from the pre-matched group in age, gender, and residential regions. After propensity score matching, the characteristics of physicians and the post-matched sample were similar.

**Table 1:** Baseline characteristics of physicians and comparison groups

**Characteristics**	**Physicians**		**General adults**					
			**Pre-matched sample**			**Post-matched sample**		
	**n = 1,686**	**%**	**n = 81,627**	**%**	***P *value**	**n** **=** **1,686**	**%**	***P *value**
Age (years)								
25–34	651	38.6	38,478	47.1	<0.001	650	38.6	0.96
35–44	619	36.7	30,997	38		621	36.8	
45–54	305	18.1	11,116	13.6		311	18.4	
55–64	111	6.6	1,036	1.3		104	6.2	
Sex								
Female	236	14	27,342	33.5	<0.001	237	14.1	0.96
Male	1,450	86	54,285	66.5		1,449	85.9	
Major diseases								
No	1,636	97	79,148	97	0.87	1,640	97.3	0.68
Yes	50	3	2,479	3		46	2.7	
Urbanicity								
Urban	1,214	72	58,372	71.5	0.11	1,215	72.1	1.00
Suburban	401	23.8	20,489	25.1		401	23.8	
Rural	71	4.2	2,766	3.4		70	4.2	
Residential region								
North	824	48.9	53,031	65	<0.001	823	48.8	0.98
Central	335	19.9	10,552	12.9		332	19.7	
South	485	28.8	16,829	20.6		485	28.8	
East	42	2.5	1,215	1.5		46	2.7	

### Utilization of imaging modalities

**Figure 1:**
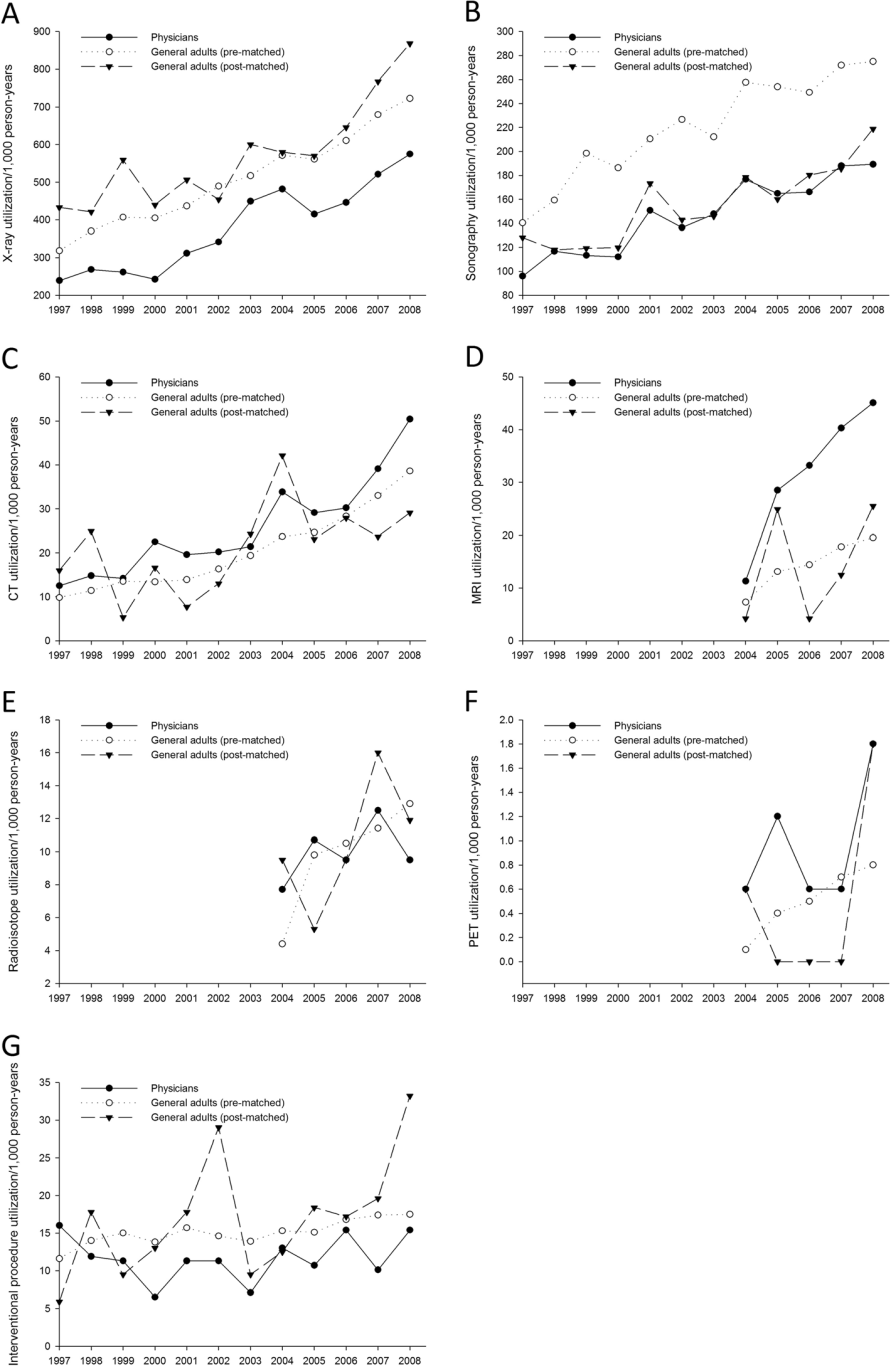
The utilization trends of (A) X-ray, (B) sonography, (C) computed tomography (CT), (D) magnetic resonance imaging (MRI), (E) radioisotope, (F) positron emission tomography (PET), and (G) interventional procedure among physicians and general adults.

The utilization trends of radiology services among physicians and the comparison groups are shown in Figure 1. The utilization rates of most radiology services increased among the physicians and the comparison groups. The utilization rates of CT and MRI were significantly higher among physicians than among the post-matched comparison group (rate ratio [RR]: 1.24, 95% confidence interval [CI]: 1.09–1.41, *P* < 0.001 for CT and RR: 2.29, 95% CI: 1.84–2.87, *P* < 0.001 for MRI) (Table 2). In contrast, the utilization rates of X-rays, sonography, and interventional procedures were significantly lower among physicians than among general adults (RR: 0.66, 95% CI: 0.65–0.68, *P* < 0.001 for X-rays; RR: 0.94, 95% CI: 0.90–0.99, *P* = 0.02 for sonography; and RR: 0.71, 95% CI: 0.60–0.83, *P* < 0.001 for interventional procedures).

**Table 2:** Utilization of radiology modalities per 1,000 person-years

**Modality**	**Physicians**	**General adults**	**Rate ratio**	**95% CI**	***P *value**
	**n** **=** **1,686**	**n** **=** **1,686**			
X-ray	379	570	0.66	0.65–0.68	<0.001
Ambulatory care	329	493	0.67	0.65–0.69	<0.001
Inpatient care	50	77	0.65	0.60–0.70	<0.001
Sonography	146	156	0.94	0.90–0.99	0.02
Ambulatory care	138	147	0.94	0.89–0.99	0.02
Inpatient care	8	9	0.89	0.72–1.11	0.28
Computed tomography	26	21	1.24	1.09–1.41	0.001
Ambulatory care	21	18	1.17	1.01–1.35	0.03
Inpatient care	5	3	1.66	1.19–2.31	0.002
Magnetic resonance imaging	32	14	2.29	1.84–2.87	<0.001
Ambulatory care	29	10	2.90	2.26–3.77	<0.001
Inpatient care	3	4	0.74	0.42–1.27	0.25
Radioisotope scanning	10	10	1.00	0.73–1.37	1.00
Ambulatory care	8	8	1.00	0.70–1.42	1.00
Inpatient care	2	2	1.00	0.48–2.08	1.00
Positron emission tomography	0.9	0.5	2.00	0.54–9.08	0.27
Ambulatory care	0.7	0.4	2.00	0.43–12.36	0.34
Inpatient care	0.2	0.1	2.00	0.10–118	0.63
Interventional procedures	12	17	0.71	0.60–0.83	<0.001
Ambulatory care	9	14	0.64	0.535–0.78	<0.001
Inpatient care	3	3	1.00	0.69–1.45	1.00

95% CI: 95% confidence interval.

Table 3 presents the adjusted odds ratios of utilization of each imaging modality. After adjusting for age, gender, major diseases, urbanicity, and residential regions, physicians utilized more MRI (odds ratio [OR]: 2.19, 95% CI: 1.68–2.84, *P* < 0.001) but less X-rays and interventional procedures (OR: 0.85, 95% CI: 0.72–0.99, *P* = 0.04 for X-rays and OR: 0.67, 95% CI: 0.54–0.83, *P* < 0.001 for interventional procedures) in comparison to the post-matched group of general adults.

**Table 3:** Radiology utilization among physicians and general adults

**Modality**	Odds ratio*	**95% CI**	***P *value**
X-ray			
General adults	1.00		
Physicians	0.85	0.72–0.99	0.04
Sonography			
General adults	1.00		
Physicians	1.04	0.90–1.20	0.62
Computed tomography			
General adults	1.00		
Physicians	1.14	0.95–1.37	0.16
Magnetic resonance imaging			
General adults	1.00		
Physicians	2.19	1.68–2.84	<0.001
Radioisotope scanning			
General adults	1.00		
Physicians	0.97	0.66–1.42	0.87
Positron emission tomography			
General adults	1.00		
Physicians	1.57	0.39–6.40	0.53
Interventional procedures			
General adults	1.00		
Physicians	0.67	0.54–0.83	<0.001

*Values adjusted for age, sex, major diseases, urbanicity, and residential regions; 95% CI: 95% confidence interval.

### Utilization stratified by coexistence of major diseases

Table 4 presents the utilization rates of radiology modalities per 1,000 person-years stratified by coexisting major diseases among physicians and post-matched general adults. When compared to general adults without major diseases, physicians without major diseases used more CT and MRI (RR: 1.23, 95% CI: 1.06–1.43, *P* = 0.004 for CT and RR: 2.07, 95% CI: 1.65–2.60, *P* < 0.001 for MRI) but less X-rays, sonography, and interventional procedures (RR: 0.65, 95% CI: 0.63–0.67, *P* < 0.001 for X-rays; RR: 0.93, 95% CI: 0.88–0.98, *P* = 0.009 for sonography; and RR: 0.69, 95% CI: 0.58–0.82, *P* < 0.001 for interventional procedures). Physicians with major diseases used more MRI (RR: 3.17, 95% CI: 1.47–7.57, *P* = 0.001) but less X-rays and radioisotope scanning (RR: 0.78, 95% CI: 0.70–0.87, *P* < 0.001 for X-rays; RR: 0.44, 95% CI: 0.22–0.87, *P* = 0.01 for radioisotope scanning) than general adults with major diseases.

**Table 4:** Utilization of radiology modalities per 1,000 person-years stratified by major diseases

**Modality**	**No major diseases**					**Major diseases**				
	**Physicians**	**General adults**	**Rate ratio**	**95% CI**	***P *value**	**Physicians**	**General adults**	**Rate ratio**	**95% CI**	***P *value**
	**n** **=** **1,636**	**n** **=** **1,640**				**n** **=** **50**	**n** **=** **46**			
X-ray	359	549	0.65	0.63–0.67	<0.001	1042	1330	0.78	0.70–0.87	<0.001
Sonography	138	148	0.93	0.88–0.98	0.009	432	435	0.99	0.83–1.19	0.94
Computed tomography	21	17	1.23	1.06–1.43	0.004	178	169	1.06	0.79–1.41	0.69
Magnetic resonance imaging	29	14	2.07	1.65–2.60	<0.001	124	39	3.17	1.47–7.57	0.001
Radioisotope scanning	9	7	1.30	0.91–1.87	0.14	56	126	0.44	0.22–0.87	0.01
Positron emission tomography	0.12	0	NA	NA	NA	28	17	1.65	0.41–7.50	0.47
Interventional procedures	11	16	0.69	0.58–0.82	<0.001	37	60	0.61	0.34–1.08	0.08

NA: not analyzed because no patients in the control received positron emission tomography; 95% CI: 95% confidence interval.

### Predictors of utilization of MRI

**Figure 2:**
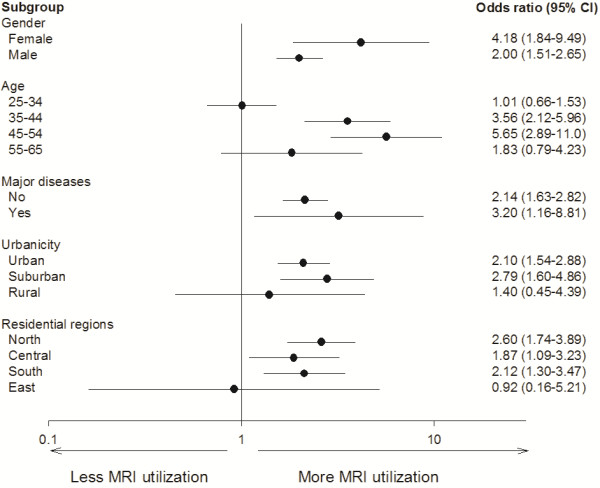
Subgroup analysis of utilization of MRI.

The predictors of utilization of MRI are shown in Table 5. The occupation of physician (OR: 2.19, 95% CI: 1.68–2.84, *P* < 0.001), age (OR: 1.63, 95% CI: 1.02–2.58, *P* = 0.04 for age 55–65 compared to age 25–34), and coexistence of major diseases (OR: 4.33, 95% CI: 2.68–7.00, *P* < 0.001) were significant predictors of utilization of MRI. Subgroup analyses showed that physicians used more MRI than general adults in most of the subgroups analyzed (Figure 2).

**Table 5:** Multiple logistic regression for prediction of usage of MRI

**Variables**	**Odds ratio**	**95% CI**	***P *value**
Physician			
No	1.00		
Yes	2.19	1.68–2.84	<0.001
Age (years)			
25–34	1.00		
35–44	0.90	0.66–1.22	0.49
45–54	1.36	0.96–1.91	0.08
55–65	1.63	1.02–2.58	0.04
Sex			
Female	1.00		
Male	0.96	0.66–1.39	0.83
Major diseases			
No	1.00		
Yes	4.33	2.68–7.00	<0.001
Urbanicity			
Urban	1.00		
Suburban	1.05	0.77–1.42	0.77
Rural	1.20	0.66–2.18	0.56
Residential region			
North	1.00		
Central	1.32	0.94–1.85	0.11
South	1.08	0.79–1.47	0.62
East	1.28	0.59–2.77	0.53

MRI: magnetic resonance imaging; 95% CI: 95% confidence interval.

### Predictors of utilization of X-ray

The predictors of utilization of X-ray are shown in Table 6. The occupation of physician (OR: 0.85, 95% CI: 0.72–0.99, *P* = 0.04) was a significant predictor of less utilization of X-ray. Other significant predictors included age (OR: 0.81, 95% CI: 0.68–0.97, *P* = 0.02 for age 35–44 compared to age 25–34; OR: 1.38, 95% CI: 1.09–1.75, *P* = 0.008 for age 45–54 compared to age 25–34; and OR: 2.03, 95% CI: 1.34–3.07, *P* = 0.001 for age 55–65 compared to age 25–34), coexistence of major diseases (OR: 29.21, 95% CI: 4.06–210.19, *P* = 0.001), and living in the central and east regions of Taiwan (OR: 1.27, 95% CI: 1.01–1.60, *P* = 0.04 for living in the central region compared to living in the north and OR: 1.87, 95% CI: 1.04–3.39, *P* = 0.04 for living in the east region compared to living in the north).

**Table 6:** Multiple logistic regression for prediction of usage of X-ray

**Variables**	**Odds ratio**	**95% CI**	***P *value**
Physicians			
No	1.00		
Yes	0.85	0.72–0.99	0.04
Age (y)			
25–34	1.00		
35–44	0.81	0.68–0.97	0.02
45–54	1.38	1.09–1.75	0.008
55–65	2.03	1.34–3.07	0.001
Sex			
Female	1.00		
Male	0.83	0.66–1.05	0.13
Major diseases			
No	1.00		
Yes	29.21	4.06–210.19	0.001
Urbanicity			
Urban	1.00		
Suburban	0.94	0.77–1.14	0.54
Rural	1.24	0.80–1.93	0.33
Residential region			
North	1.00		
Central	1.27	1.01–1.60	0.04
South	1.03	0.85–1.24	0.80
East	1.87	1.04–3.39	0.04

95% CI: 95% confidence interval.

### Predictors of utilization of interventional procedures

The predictors of utilization of interventional procedures are shown in Table 7. Physicians used significantly less interventional procedures (OR: 0.67, 95% CI: 0.54–0.83, *P* < 0.001). Age (OR: 1.48, 95% CI: 1.11–1.97, *P* = 0.007 for age 45–54 compared to age 25–34 and OR: 2.46, 95% CI: 1.70–3.55, *P* < 0.001 for age 55–65 compared to age 25–34), male sex (OR: 1.46, 95% CI: 1.03–2.08, *P* = 0.04), and coexistence of major diseases (OR: 3.16, 95% CI: 2.00–4.98, *P* < 0.001) were significant predictors of utilization of interventional procedures.

**Table 7:** Multiple logistic regression for prediction of usage of interventional procedures

**Variables**	**Odds ratio**	**95% CI**	***P *value**
Physicians			
No	1.00		
Yes	0.67	0.54–0.83	<0.001
Age (y)			
25–34	1.00		
35–44	0.87	0.67–1.13	0.30
45–54	1.48	1.11–1.97	0.007
55–65	2.46	1.70–3.55	<0.001
Sex			
Female	1.00		
Male	1.46	1.03–2.08	0.04
Major diseases			
No	1.00		
Yes	3.16	2.00–4.98	<0.001
Urbanicity			
Urban	1.00		
Suburban	1.01	0.77–1.32	0.94
Rural	1.60	0.98–2.61	0.06
Residential region			
North	1.00		
Central	0.80	0.59–1.08	0.14
South	0.88	0.68–1.13	0.31
East	0.46	0.19–1.08	0.07

95% CI: 95% confidence interval.

## Discussion

The analyses revealed that the utilization rates of most radiology services increased for all groups during the study period. The rapid increase in use of CT and MRI in recent years is regarded as one of the factors responsible for growing medical costs [4]. Increases in supply-side factors have been observed to significantly increase CT and MRI utilization [8], while physician and hospital characteristics have also been shown to be associated with repeated use of CT and MRI [15,16]. However, when the physician becomes the consumer and not the provider, the complex role of the physician as agent between insurers and patients no longer exists [17].

Our results showed that utilization rates of some radiology modalities varied significantly when physicians and non-physicians were compared. The rate ratios of X-rays, sonography, and interventional procedures were significantly lower among physicians than among non-physicians. After controlling for age, gender, major diseases, urbanicity, and residential regions, the odds of utilization of MRI among physicians remained significantly higher than among non-physicians. Compared to CT, MRI is relatively safe and free of radiation exposure characteristics [18-20], and MRI can offer a definitive diagnosis faster (thereby leading to earlier treatment) than less advanced modalities like X-rays and sonography. Medical knowledge, familiarity with the health care system, and better patient-doctor communication may have been the contributory factors for the preference of MRI among physicians [21,22]. The self-prescription rate of MRI among the physicians was 0.27% in this study. Although physicians have the right to prescribe MRI, self-prescription among physicians was not a major reason for higher utilization of MRI. Additionally, differences in use of radiology modalities between physicians and non-physicians may occur because physician patients are more knowledgeable about their illnesses and prefer to receive detailed information from radiology services departments, but the differences may also reflect misuse or overuse of radiology services by physician patients. It is a limitation of our study that we cannot make this differentiation because of a lack of information on participants’ decision-making processes and because no objective measure has been developed of the value of radiology modalities in improving health.

Previous findings have indicated that physicians may treat their peers differently than their other patients, perhaps because the treating physicians feel pressure from these informed patients, also could be a sort of showing favors to colleagues [23,24]. On the other hand, they may also face greater medical scrutiny from their fellow doctors if treatments of questionable medical value are recommended. The care-seeking behavior and utilization patterns of radiological services of physicians, medically savvy consumers who are familiar with the health care system, may provide useful insights into defining the appropriate use and quality of health care.

The results of this research should be viewed in light of several limitations. First, since physicians were identified as those who had “physician” as their recorded occupation in the medical personnel registry, physicians who were not registered as practicing would be classified as general adults. However, this misclassification bias would underestimate our findings and lead to the results here being more conservative than they really are. Second, as mentioned previously, this study might suffer from certain inherent limitations because of the use of administrative data, which do not provide information on the reasons why radiology modalities were chosen. Third, it is impossible to determine from our results whether the utilization of radiology modalities of the physicians or non-physicians represents more appropriate care, because there is no objective measure of the value of radiology modalities in improving health. Fourth, the external validity of the results may be a concern. This study included only physicians between the ages of 25 and 65 and was conducted in a national health insurance setting with nearly universal access. The results may not be applicable to countries with different health care systems.

## Conclusions

This population-based study revealed that patients who are physicians are more likely to use MRI compared to non-physicians, but less likely to use some other modalities. These different utilization patterns of radiology modalities between physicians and non-physicians provide valuable information for the continuing discussion of the appropriate use of limited health care resources.

## Consent

Written informed consent was obtained from the patient for the publication of this report and any accompanying images.

## Abbreviations

CT: Computed tomography; MRI: Magnetic resonance imaging; PET: Positron-emission tomography; NHI: National health insurance; SES: Socioeconomic status; RR: Rate ratio; OR: Odds ratio; CI: Confidence interval; USD: United States Dollar.

## Competing interests

The authors declare that they have no competing interests.

## Authors’ contributions

CYW contributed to the study design, statistical analysis, interpretation of data, and writing of the manuscript. HYH contributed to acquisition of data, study design, data analysis, and interpretation of data. LC contributed to statistical analysis and revision of the manuscript. NH contributed to study design, interpretation of data, and revision of the manuscript. YJC contributed to study design, interpretation of data, and revision of the manuscript. CPL contributed to statistical analysis, interpretation of data, writing the manuscript, and revising the manuscript. All authors have given final approval of the version to be published.

## Pre-publication history

The pre-publication history for this paper can be accessed here:

http://www.biomedcentral.com/1472-6963/13/284/prepub
